# Homœopathic Prescribing

**Published:** 1889-03

**Authors:** Julius G. Schmitt

**Affiliations:** Rochester, N. Y.


### HOMŒOPATHIC PRESCRIBING.

Julius G. Schmitt, M. D., Rochester, N. Y.

Lately there has been a great deal said and written about using books at the bedside of the patient, and with some physicians, who would not open a book in presence of their patient for fear of betraying ignorance, the belief seems to have taken possession that those who use books do not rely upon their memory at all, but, if they are called to a patient, sit down and study their whole materia medica, and if it were to prescribe but Aconite in a case of fever with chilliness when moving or uncovering and anxious tossing about.

The undersigned, a prescriber from materia medica and repertories at the bedside of the patient, should like to have a little to say in this matter.

Starting out on my daily rounds of visits, I carry in my satchel, like most of the followers of Hahnemann in this city, Hering's *Materia Medica*, Lippe's *Repertory*, and Bell's book on diarrhœa, not with the intention of using them in every ordinary case that may have to be prescribed for, but to be in readiness for the *extraordinary* demands that are often made on the prescriber.

A case of uterine hemorrhage, diphtheria, croup, pneumonia, scarlet fever, gastralgia, colic, etc., will seldom require us to have recourse to the books, since the more acute and dangerous a disease, the more characteristic are generally the symptoms, and a physician who has often consulted his books will be familiar enough with their contents to recognize the called-for remedy. Here, also, often so-called "intuition" comes in, which, however, should more properly be designated "unconscious cerebration." But this action can only take place in a brain where there is some storage.

Not long ago Dr. J. A. Biegler saw a case with me that had baffled my endeavors so far. The morning we met at the patient's her symptoms had cleared up remarkably, and were as follows :

Old lady, about sixty-two years of age, complains of heaviness of lower extremities, so that she can only walk by the help of a cane ; worse evenings. Itching of canthi, better from rubbing. Oppression of chest, worse lying down. Borborygmi in left hypochondrium ; red sand in the urine.

We both had our satchels with books, but did not think of looking at them when there was Lycopodium so plainly indicated.

A child, twenty-two months old, getting his last bicuspids, is fretting day and night, wants to be carried all the time. Cough with gagging, especially when excited; mouth hot, palms of hands hot. Afraid of falling, when mother lays him down in the cradle. Now, who ever claiming to be a student of materia medica, could prescribe anything else but Borax?

Examples after examples could be cited, where even the book prescriber never resorts to his books, and yet he often meets cases where he would do a great deal of harm by wrong prescriptions were it not for the books he has ready for use. Permit me to quote again from practice.

A girl, ten years old, sick with typhoid fever for three weeks, presents the following condition: Paralysis of left side of face, loss of speech, grinding of teeth as if they were going to be crushed to pieces. Stiffness, pain and tenderness of right side of neck and nape. Vomiting of brown fluid every evening from six to eight o'clock. Pulse 120, temperature 101.4.

Lippe's *Repertory* helps us in the following manner:

*Grinding of teeth* (which was the latest symptom) we find under: Acon., Ant., Apis, Arn., *Ars.*, Aur., Baryt-c., Canth., Caustic, Cham., Cic., Cin., Coff., Con., *Hyosc.*, Igt., Lyc., Merc., Phosph., Plb., Pod., Sec-c., *Stram.*, *Verat-alb.*

*Loss of power of speech:* Ars., *Caustic.*, *Cic.*, Con., *Hyosc.*, *Merc.*, *Plb.*, *Stram.*, *Verat* (and others, which, however, have *not* the grinding of the teeth).

*Stiffness of nape of neck:* Ars., *Caustic.*, Merc., Verat-alb. Brownish vomiting we only find under Ars. and Bismuth, but Arsenic had been given, especially on account of the periodicity of the vomiting, but without any response.

Paralysis of half of the face is a symptom belonging to Caustic. and Graphites. Under Causticum, we read in Hering's *Materia Medica*, "Heat from six to eight P. M.;" the question arose: Could not vomiting, appearing at this time, be also cured by Causticum, that has the heat at this time, and which covers all the other symptoms? Such analogies often having proved correct. Causticum was prescribed and cured the case.

This patient lived half an hour's ride away from my office, and it seemed to me a great saving of time to have my books along. Besides, the above work was done in ten minutes.

Mr. Tr., twenty-eight years old, has had intermittent fever while in the Prussian army five years ago, which, of course, had been suppressed by Quinine. He was seen at half-past eight

P. M., and presented the following symptoms: Chill at three P. M., without thirst, with painful swelling of varices; at five P. M., heat with thirst and chilliness from uncovering; at half-past seven P. M., perspiration with general alleviation of symptoms. Now, I was not prepared to prescribe then and there from memory for the uncommon and peculiar symptom "*of painfulness and swelling of varicose veins during chill,*" and there are many remedies that have chill at three P. M., thirstlessness during chill, and thirst during heat. Therefore, Allen's book on intermittent fever, second edition, which had been taken along for this occasion, was consulted. Five minutes sufficed to find the correct remedy, viz.: Chinium sulph., and one dose of the CM potency cured right away; there has been no repetition of the fever since.

In my practice almost every case is taken down in writing, according to the rules of Hahnemann, and it is astonishing how often a seemingly simple case turns into a hard nut when proper care is taken to elicit every symptom. This procedure has been such a help to me in correct prescribing that I cannot understand how any man can undertake the cure of any case, especially, however, of a chronic one, without following this dictum of the master, who always knew what he was about. And yet there are many true followers of Hahnemann who never think of taking down a case in writing; if they ever would try it, they would find it to be a great saving of time, although I know they claim just the contrary.

But, even if it were so, shall time play any consideration when we are engaged in curing the suffering sick? It is the mongrel that flies from patient to patient, and is satisfied with a trifling compensation for the effort of jumping in and out of his carriage, for his legs certainly suffer more than his brain.

